# Ultra-thin rapeseed oil-assisted femtosecond laser etching on quartz glass microgrooves

**DOI:** 10.1016/j.isci.2025.111921

**Published:** 2025-02-01

**Authors:** Jing Liu, Yimin Feng, Mian Zheng, Shangkai Chen, Pengran Wang, Mengdan Zhao, Zhaoxian Huang, Ming Li

**Affiliations:** 1 College of Computer Science, South-Central Minzu University, Wuhan 430074, China; 2 China Ship Development and Design Center, Wuhan 430064, China; 3 Bayannur Power Supply Company, Inner Mongolia Power (Group) Co. LTD, Bayannur, Inner Mongolia 015000, China

**Keywords:** Optics, Laser, Materials science

## Abstract

Liquid-assisted methods can effectively suppress thermal effects, such as recrystallization, micro-cracks, and edge collapses of hard and brittle materials in femtosecond laser processing in air. However, the small and numerous bubbles generated by water solvents seriously affect laser processing efficiency. We have investigated ultra-thin rapeseed oil-assisted (approximately 58μm thickness) femtosecond laser processing of quartz glass microgrooves. Experimental results show that rapeseed oil-assisted laser ablation reduces the adverse effects of bubbles through the bubble coalescence effect but also helps to discharge the bottom debris via the formed micro-jet. According to the ionization model with the Drude equation, the laser intensity to reach the damage threshold of quartz in rapeseed oil is lower than that in air. Based on heat conduction theory, rapeseed oil can effectively reduce the quartz glass’s temperature gradient. The ultra-thin rapeseed oil-assisted femtosecond laser etching method has strong potential and important practical significance in etching glass material.

## Highlights

• Rapeseed oil-assisted laser ablation mitigates adverse effects of bubbles via coalescence
• Rapeseed oil-assisted laser ablation aids in debris removal via formed micro-jets
• Damage threshold of quartz lower in rapeseed oil vs. air, based on Drude ionization model
• Rapeseed oil reduces quartz glass temperature gradient based on Fourier heat conduction theory

## Graphical abstract

**Figure:**
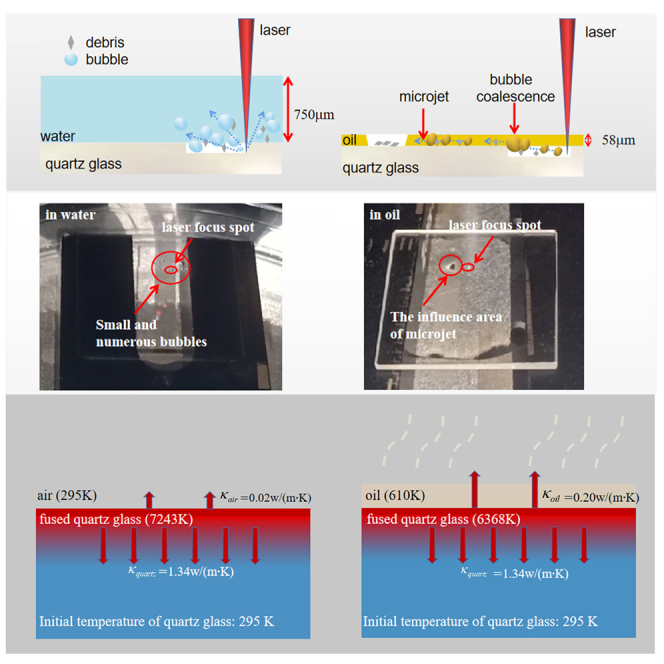

## Introduction

Transparent brittle materials,^1^ including sapphire, quartz glass, and diamond,^2^ are known as the most potential photoelectric materials with high resistivity, perfect insulation performance, and transparency, having wide application prospects in biology,^3^^,^^4^^,^^5^ integrated circuit interconnection, and other technical fields.^6^^,^^7^ However, these properties also make them difficult to process mechanically or chemically. It is a long-time technical challenge to find suitable engineering tools to fabricate devices and structures simply and precisely on these transparent hard substrates, especially at the micro-scale. With the advent of various state-of-the-art technologies, femtosecond laser micromachining technology^8^^,^^9^^,^^10^ with the advantages of high precision and low thermal effect has been commonly used for drilling, cutting, and structuring in micro-channels,^11^ micro-holes,^12^ and lab-on-a-chip.^13^ However, a few problems such as recast layer, ablative debris re-deposition, phase transition, and cracks will be encountered.^14^ Timely removal of etched debris and excess heat is essential for high-quality laser micro- and nano-machining. Compared to direct laser ablation, liquid-assisted laser etching^15^ has been proven to be an efficient method to reduce or even eliminate heat-affected zones and localized mechanical stress and quickly discharge debris deposited.^16^ The different composite methods of liquid and laser could be mainly divided into water-guided laser machining,^17^ water jet-assisted laser machining,^18^ and underwater laser ablation technology.^19^ Nevertheless, the disadvantage of liquid-assisted laser etching is that a large number of bubbles^20^^,^^21^ will be generated due to plasma expansion,^22^ and these bubbles^23^ are mainly concentrated in the etching area, leading to low energy efficiency and poor surface quality.

When in a liquid-assisted laser ablation process, a large number of cavitation bubbles originate from the high-temperature and high-pressure plasma induced by high-energy laser pulses. Motivated by the bubble coalescence phenomenon, in which two or more gas bubbles in a liquid medium collide and form one larger bubble,^24^ we utilize bubble coalescence and liquid convection to reduce the impact of bubbles on the etching region. The greater the viscosity, the greater the bubble spacing^25^ and the longer the coalescence time.^26^ At room temperature, the viscosity of rapeseed oil is 65 cP while the viscosity of water is 1 cP. Hence, rapeseed oil is selected as an auxiliary liquid. In addition, the thickness of the auxiliary liquid is also an important factor affecting the processing. Guo Yang^2^ found that a continuous and directional high-speed micro-jet has been generated when laser-induced primary cavitation bubbles asymmetrically collapse sequentially under a critical ultra-thin liquid layer, which helps to remove secondary bubbles and ablative debris in time.

In this paper, we have investigated the effect of ultra-thin rapeseed oil (about 58μm thickness) used to assist the laser processing of quartz glass microgrooves. We will analyze laser etching parameters (laser pulse energy, scanning speed, and scanning times) correlative to the geometrical parameters of glass microgrooves. Moreover, the evolution of the electron density of quartz in air and rapeseed oil is established based on the ionization model with Drude equation,^27^^,^^28^ and the transient heat conduction of quartz glass is analyzed by Fourier heat conduction theory.^29^ We found that laser etching in the ultra-thin layer of rapeseed oil has three advantages. It (1) improves the surface quality and discharges the bubbles and the bottom debris in time via the formed directional micro-jet, (2) reduces the damage threshold of the quartz glass, and (3) reduces the temperature gradient of the quartz glass surface.

## Results

### Experimental setup

**Figure 1:**
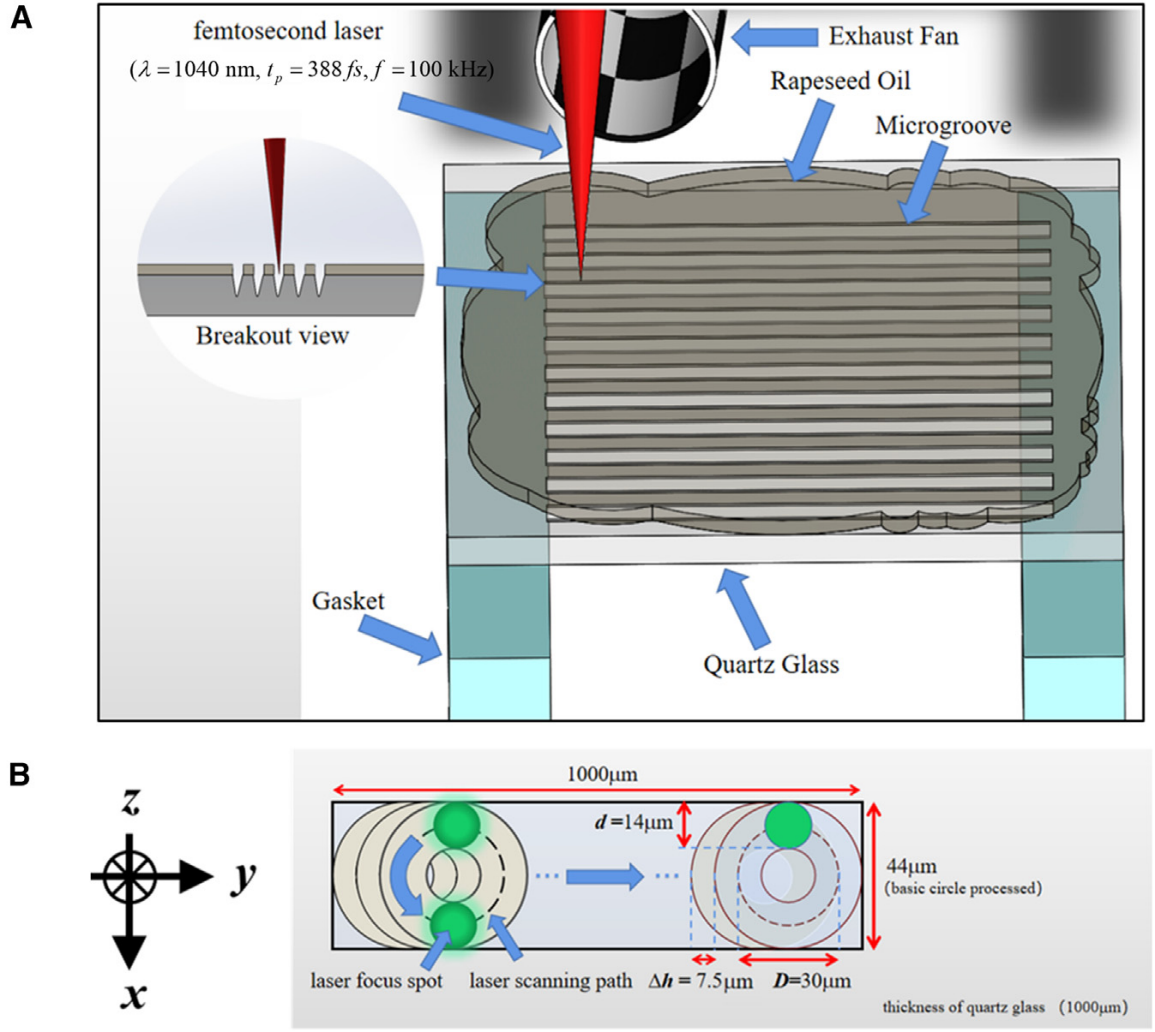
Laser etching diagram and drilling path diagram (A) Experimental schematic diagram of femtosecond laser processing. (B) The CAD drawing of the drilling process.

The femtosecond laser processing equipment used in the experiment is shown in Figure 1A. The details can be found in Figure S1 (supplemental information). The center wavelength of the femtosecond laser is 1,040nm, the pulse width is 388fs, and the repetition rate is 100kHz. The preset etching path of computer-aided design (CAD) drawing software is shown in Figure 1B. The etching path is set as an array circle diameter D of 30μm, with an interval distance d of 7.5μm.

In the experiments, 0.02ml of rapeseed oil is dropped onto the ultraviolet grade fused silica crystal (JGS1) quartz glass surface by a needle. The photo outline (85.64%) of the rapeseed oil coverage area is estimated ($342.56\;{\text{mm}}^{2}$), and the thickness of the thin layer of rapeseed oil is about 58μm. And the exhaust fan will be turned on to pump away the smoke. After etching, the sample is wiped with a dust-free mirror paper containing absolute alcohol and placed in the ultrasonic machine for 60 s to remove the rapeseed oil and debris.

**Figure 2:**
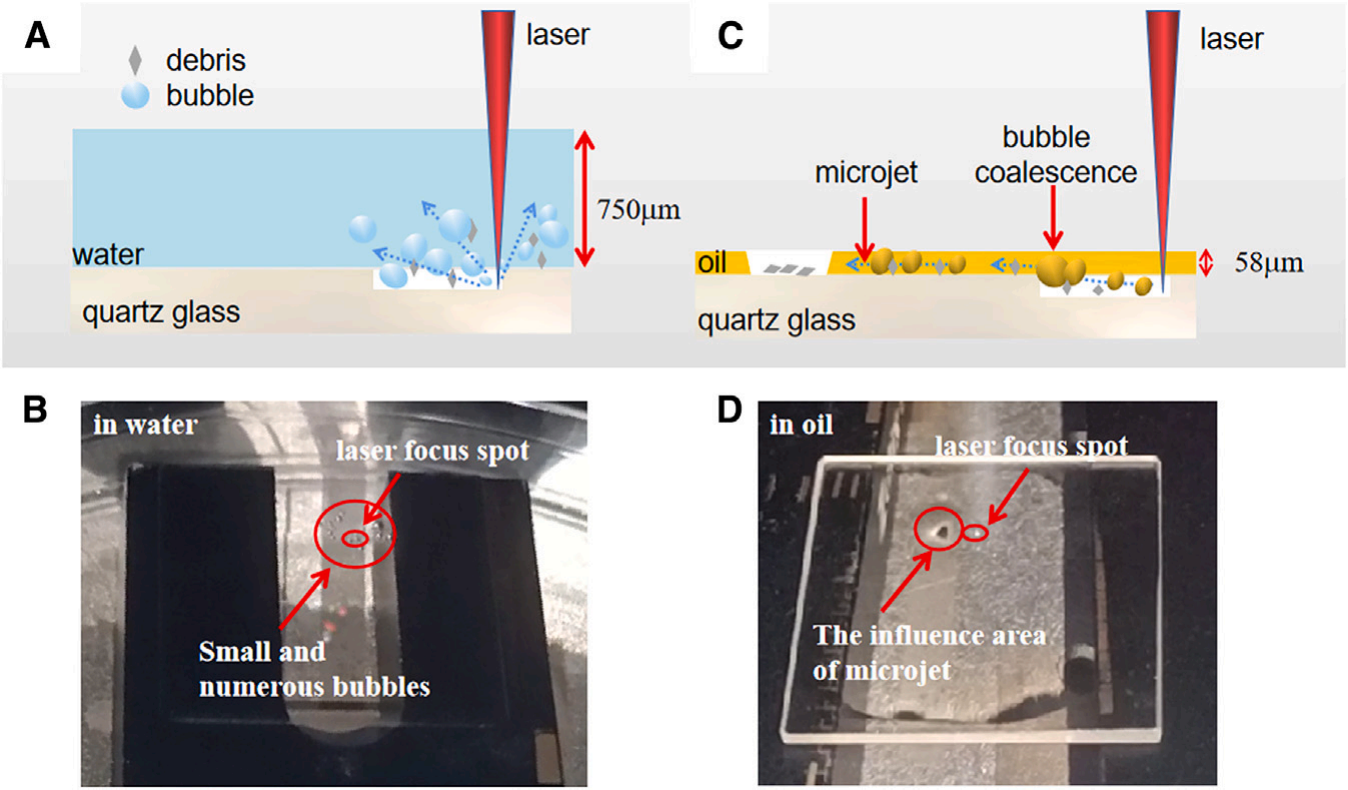
Schematic and experiment diagrams of laser etching quartz glass in water and in rapeseed oil at $E_{p}=10.6\;\mu J$ (A) Schematic diagram of laser etching quartz glass in water. (B) Experiment diagram of laser etching quartz glass in water. The thickness of the water film layer is 750μm. (C) Schematic diagram of laser etching quartz glass in rapeseed oil. (D) Experiment diagram of laser etching quartz glass in rapeseed oil. The thickness of the rapeseed oil film layer is 58μm.

We added a pre-experiment to verify the bubble coalescence when the sample was immersed in both water and rapeseed oil. During the comparative experiments, a water droplet of 0.3 mL was dispensed onto a 20 by 20 mm square substrate, completely covering it and resulting in an average film thickness of approximately 750 μm. The high surface tension of water poses a significant challenge in reducing the thickness of liquid films.^30^ As a result, there is a divergence in the thicknesses between the oil and water films. Figures 2A and 2B show the schematic and experiment diagrams of laser etching quartz glass covered by water. In Figures 2B and 2A, multitude of bubbles are generated by laser etching in water and dispersed around the processing region. The poor bubble coalescence ability of water leads to the dispersion of bubbles in the aqueous solution. This behavior is attributed to the fact that water, having a low surface tension, does not facilitate the merging of bubbles. Furthermore, due to temperature gradients around the etching region, liquid convection is intensified, causing an increase in the flow of bubbles. This convection significantly impacts the energy utilization efficiency and the uniformity of the etched material’s quality. The localized heating from the laser can lead to thermal gradients that drive fluid motion, which in turn affects bubble dynamics and can lead to non-uniform etching patterns. Owing to the large film thickness, the formation of a micro-jet is challenging.^31^ The bubble behavior can be further elucidated by referring to Video S1, which provides a visual representation of these dynamics.

Video S1. The laser etching experiment of quartz glass was conducted in water at $E_{p}=10.6\;\mu J$ (S1 water.mp4)

Figures 2C and 2D show the schematic and experiment diagrams of laser etching quartz glass covered by rapeseed oil (about 58μm thickness). In contrast to the scenario presented in Figure 2B, Figure 2D illustrates a significant difference with the rapeseed oil environment. The bubbles generated in the rapeseed oil medium exhibit a coalesced behavior, forming a continuous and directional high-speed micro-jet when the laser-induced primary cavitation bubbles asymmetrically collapse sequentially under the critical ultra-thin liquid layer condition.^32^^,^^33^ This micro-jet of bubbles, represented by the black area in Figures 2D and Video S2, is directed away from the processing area. This directional movement of the bubble micro-jet is crucial as it actively removes the bubbles from the processing zone, thereby reducing the interference with the laser beam and improving the etching process.

Video S2. The laser etching experiment of quartz glass was conducted in rapeseed oil at $E_{p}=10.6\;\mu J$ (S2 rapeseed oil.mp4)

### Experimental results and analysis

**Figure 3:**
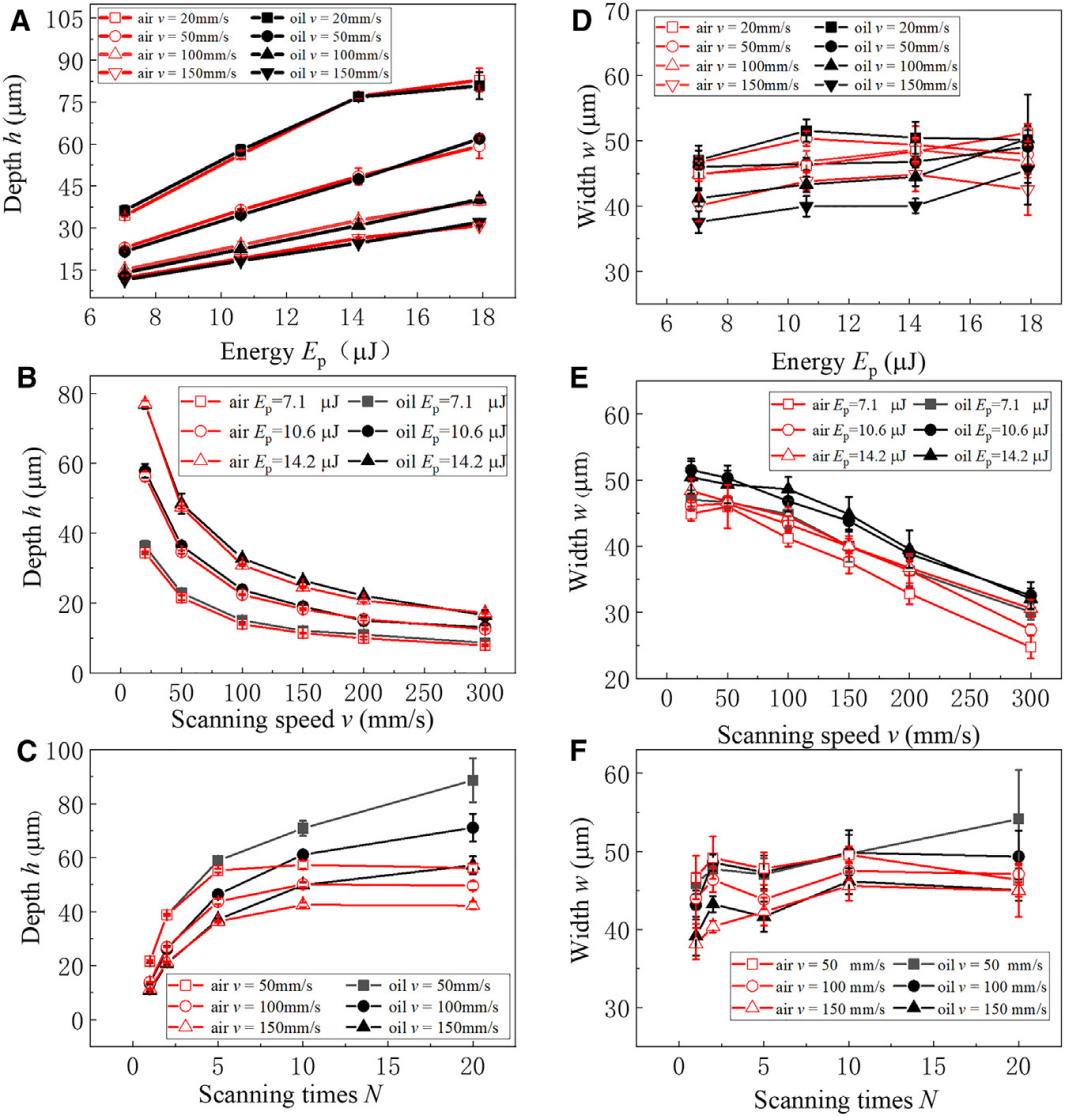
Comparison of the depth h and the width w of the microgroove etched by the femtosecond laser immersed in air and rapeseed oil (A) Sketch of $E_{p}∼h$. (B) Sketch of v∼h. (C) Sketch of N∼h. (D) Sketch of $E_{p}∼w$. (E) Sketch of v∼w. (F) Sketch of N∼w.

Figure 3 displays the comparison of the depth h and width w of the microgroove varying with the laser single pulse energy $E_{p}$, the scanning speed *v*, and the scanning time N etched by the femtosecond laser immersed in both air and rapeseed oil. In Figure 3A, when N=1, the depth *h* of microgroove increases as $E_{p}$ increases both in air and rapeseed oil. And the depths of etched microgrooves are similar. In Figure 3D, both in rapeseed oil and in air, the width w of the microgroove is not sensitive to $E_{p}$, which basically fluctuates around 40μm to 50μm. The width is mainly determined by the laser spot radius and laser processing methods. As shown in Figure 3B, the depth h of the microgroove gradually decreases with the increase of v both in air and in rapeseed oil when N=1. This is primarily owing to an increase in the scanning speed as the energy density of the laser acting on quartz glass decreases. The depth h of the microgroove gradually tends to stabilize with increasing v. In Figure 3E, the width w of the microgroove reduces as v increases both in air and in rapeseed oil. It is noteworthy that the depth h and width w of the microgroove in rapeseed oil are slightly higher than those in air. This phenomenon indicates that, by using rapeseed oil, the energy required to cause irreversible damage to the sample surface can be significantly reduced.

Furthermore, at the condition of $E_{p}=7.1\;\mu J$, the impact of the N on the depth h and width w of quartz glass microgroove is respectively studied, displayed in Figures 3C and 3F. In Figure 3C, when N<5, the depth h of the microgroove gradually increases as N increases both in air and in rapeseed oil. There is little difference in the depth of the microgrooves under different etching mediums. But, when N≥5, the depth of the microgroove in rapeseed oil is significantly greater than that in air. When in air, with the increase of N, the residue at the bottom of the microgroove accumulates to a certain height, resulting in more recast layers that hinder the laser beam. It slows down the depth of the microgroove, and the depth h finally reaches a saturation. In comparison, the depth of the microgroove in rapeseed oil gradually increases with the increase of N. The etching process in rapeseed oil has higher processing efficiency compared with air. There are mainly particular two reasons. The first is that the debris can be discharged in time, which is mainly because the impact force generated from the micro-explosion inside quartz glass and the driving force originating from the bubble rupture can drive the flow of liquid.^34^ The second is that rapeseed oil can absorb the laser energy to cause damage to the sample surface and also serve as a heat dissipation medium, reducing re-melted layer. As presented in Figure 3F, with the increase of N, the width w of the microgroove fluctuates around 40μm to 50μm in both air and rapeseed oil. It implied that N has less impact on the width of the quartz glass microgroove in both rapeseed oil and air.

### Surface morphology of quartz glass etched in air and rapeseed oil

**Figure 4:**
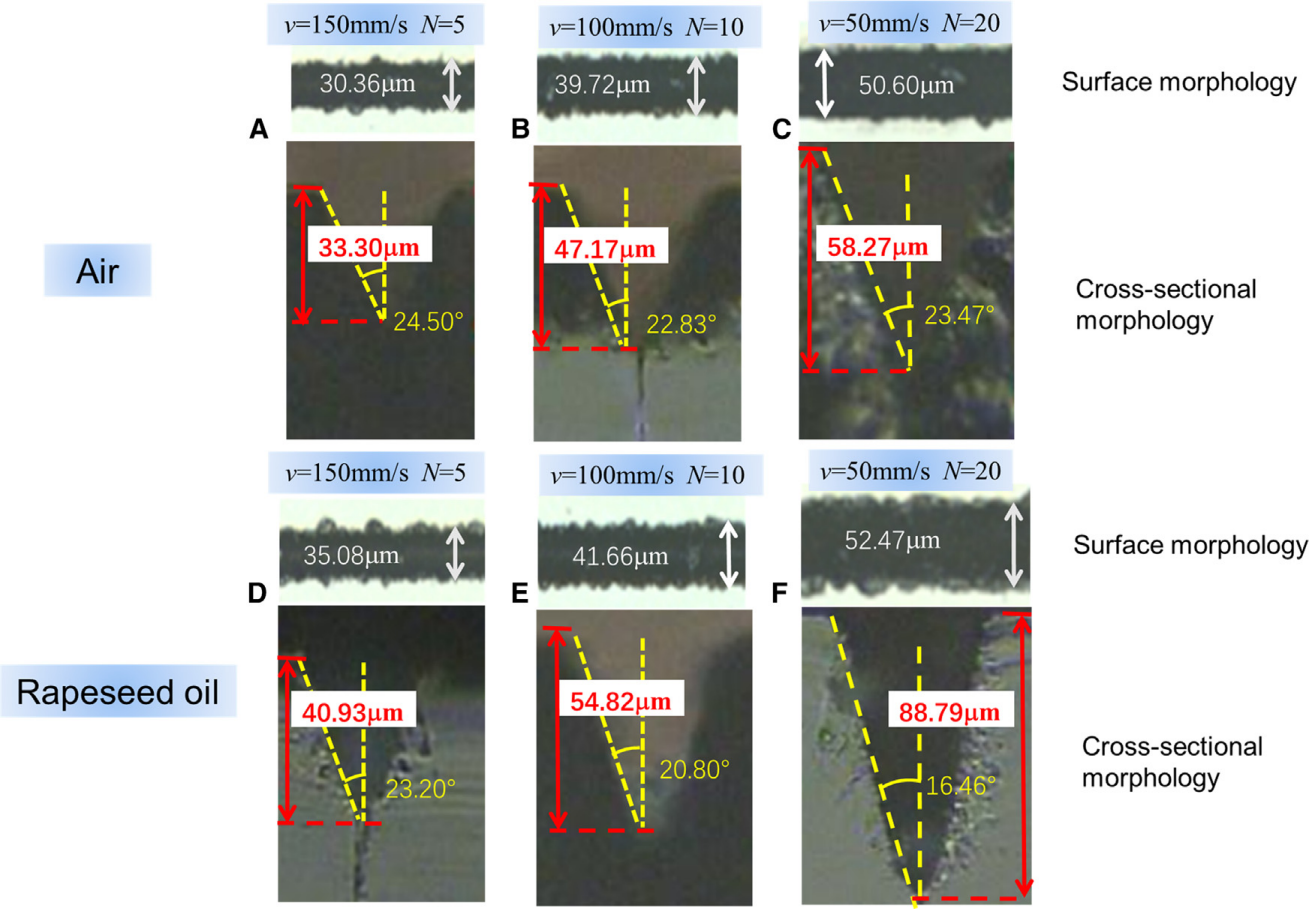
When $E_{p}=7.1\;\mu J$, the surface and cross-sectional morphology of quartz glass microgrooves at different velocity *v* and scanning times *N* in air and rapeseed oil (A–C) In the air, (A) v=150mm/s,N=5, (B) v=100mm/s,N=10, and (C) v=50mm/s,N=20. (D–F) In rapeseed oil, (D) v=150mm/s,N=5 (F) v=100mm/s,N=10 (E) v=50mm/s,N=20.

Under the experimental condition of $E_{p}=7.1\;\mu J$, the surface and cross-sectional morphology of quartz glass microgrooves at different velocity *v* and scanning times N is presented in Figure 4. In the air, when (1) v=150mm/s,N=5; (2) v=100mm/s,N=10; and (3) v=50mm/s,N=20, the widths of microgroove are 30.36μm, 39.72μm, and 50.60μm; the tape angles of microgroove are 24.50°, 22.83°, and 23.47°; and the depths of microgroove are 33.30μm, 47.17μm, and 58.27μm, respectively, as shown in Figures 4A–4C. The aspect ratio of microgroove etched in air is 1.10, 1.19, and 1.15. At the same condition, when quartz glass is etched in rapeseed oil, the widths of the microgroove are 35.08μm, 41.66μm, and 52.47μm; the tape angles of microgroove are 23.20°, 20.80°, and 16.46°; and the depths of microgroove are 40.93μm, 54.82μm, and 88.79μm, respectively, which are observed in Figures 4D–4F. The aspect ratio of microgroove etched in rapeseed oil is 1.17, 1.32, and 1.69. The results indicate that the rapeseed oil-assisted femtosecond laser etching on quartz glass will contribute to manufacturing microgrooves with a high aspect ratio.

Comparative experimental results of quartz glass microgroove cross-sectional morphologies in air and rapeseed oil at $E_{p}=7.1\;\mu J$, v=50mm/s, and N=5 are given in Figure S2. It can be observed that there are more etching micro-cracks and carbonization in the air than in the rapeseed oil. This is attributed to the oil’s higher specific heat capacity and thermal conductivity, which facilitate the absorption and dispersion of heat away from the etching zone, thereby lowering the likelihood of material charring and enhancing the overall quality of the microgroove.

### Simulation analysis

#### Absorption characteristics

**Figure 5:**
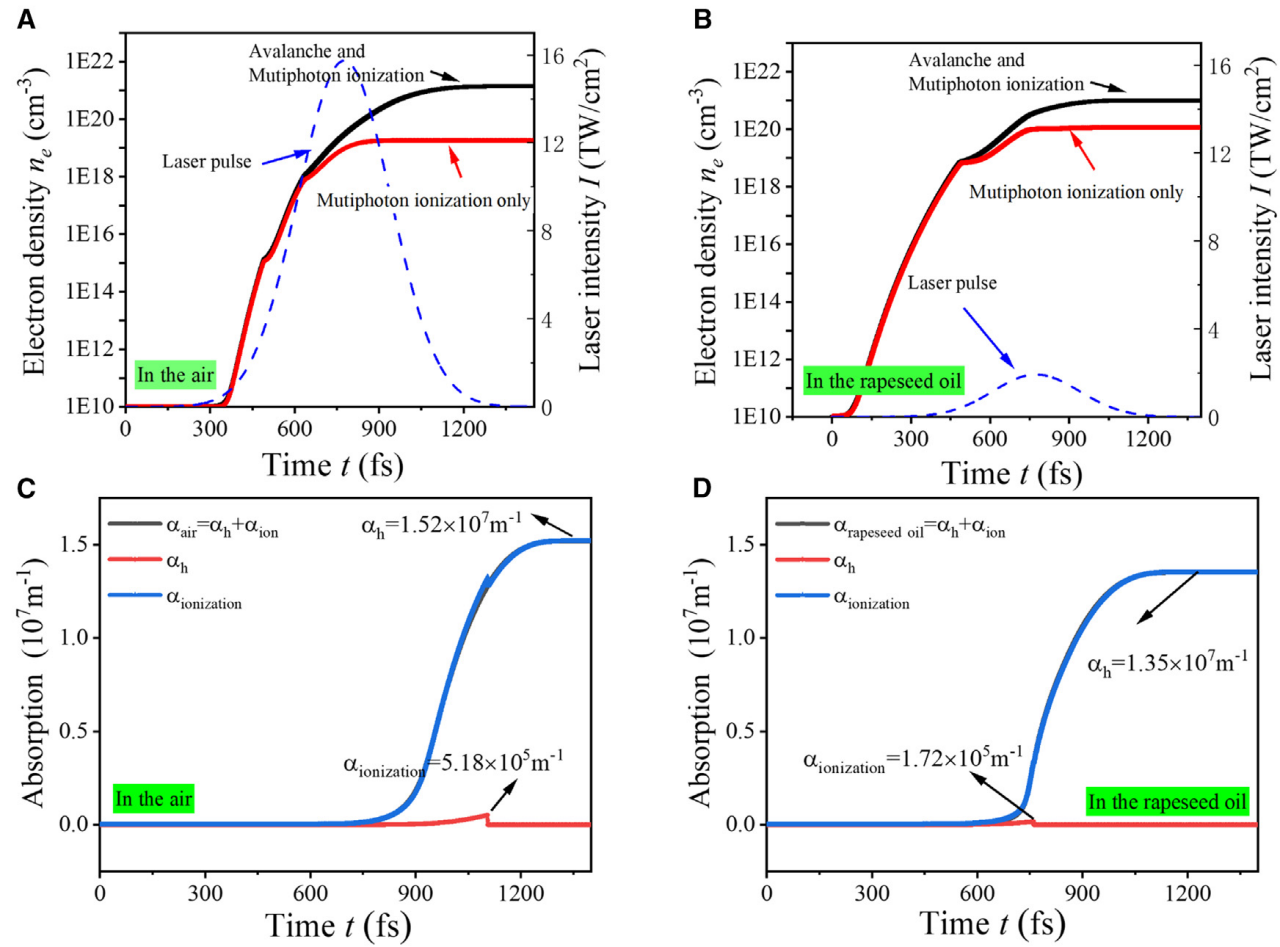
Time t dependence of the electron densities $n_{e}$ and absorption for a 1,040nm, 388fs laser pulse in the air and rapeseed oil (A) A laser fluence $F=6.5\;J/{\text{cm}}^{2}$ in the air. (B) A laser fluence $F=0.8\;J/{\text{cm}}^{2}$ in rapeseed oil. (C) Variation of total absorption coefficient, heating coefficient, and ionization coefficient in the air. (D) Variation of total absorption coefficient, heating coefficient, and ionization coefficient in rapeseed oil.

Laser energy absorption plays a crucial role in the process of laser etching. When the material absorbs energy, it generates plasma, causing local material to continuously vaporize or be stripped away, thus forming tiny holes or structures to achieve the etching effect. The algorithm detailing about the free electron density and absorption via the ionization model can be found in Kan et al.^35^ In the simulations, the band gaps of quartz glass and rapeseed oil are 8.9eV and 3.5eV, respectively, and the other main parameters are listed in Table S1. When the free electron concentration reaches $n_{e}=10^{21}\;/{\text{cm}}^{3}$, the substance is assumed to be broken down. Figures 5A and 5B show the evolution of electron density of quartz in air and rapeseed oil at the laser pulse of 388fs, 1,040nm. They demonstrated that, when the laser intensity reaches $15.3\;\text{TW}/{\text{cm}}^{2}$ and $1.19\;\text{TW}/{\text{cm}}^{2}$, the quartz and rapeseed oil begin to break down, respectively. The results illuminate that rapeseed oil requires lower light intensity to meet the standard threshold than quartz. This phenomenon was also reported by Sun et al.^36^ This also explains why the etching width of quartz in rapeseed oil is wider and deeper than that in the air under the same conditions in Figure 3. In addition, the absorption of laser energy in the air and the rapeseed oil is plotted in Figures 5C and 5D. Although the laser intensity in the rapeseed oil is significantly lower than that in air upon reaching the damage threshold of quartz, the nonlinear absorption coefficient of rapeseed oil is $1.35\times 10^{7}\;m^{-1}$, which is roughly on par with that of rapeseed oil $1.52\times 10^{7}\;m^{-1}$. It means that rapeseed oil can absorb more laser energy than air. If the incident light intensity is between the threshold of quartz and that of rapeseed oil, the quartz glass cannot be etched in air but it can be etched in rapeseed oil. The experimental outcomes substantiate this phenomenon, shown in Figure S3. At v=150mm/s, Figures S3A and S3B reveal that the microgrooves etched in air exhibit discontinuities and do not achieve the desired shape fully. In contrast, under identical conditions in rapeseed oil, complete microgroove profiles are observable. When the scanning speed v=200mm/s, the etching depth is nearly imperceptible in air, yet it is distinctly visible in oil, as shown in Figures S3C and S3D. The rapeseed oil plays a role in mitigating the thermal effects and reducing the damage threshold of the quartz glass, thus enabling a more precise and continuous etching process.

#### Dissipation characteristics

**Figure 6:**
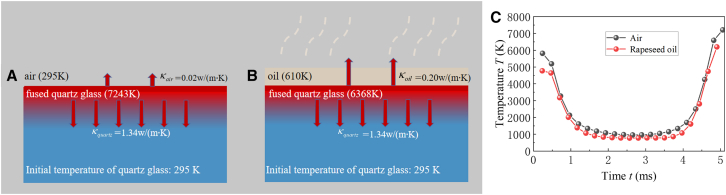
Heat transfer diagram and the temperature of quartz glass over time t in air and rapeseed oil (A) Heat transfer diagram in air. (B) Heat transfer diagram in rapeseed oil. (C)Time t dependence of the temperature of quartz glass in air and rapeseed oil. The temperature probe is located at the position [15μm,0,0].

As a liquid layer, rapeseed oil also plays a significant role in dissipating energy. According to Fourier heat conduction theory, the three-dimensional nonlinear transient heat conduction differential equation in laser processing can be established. The details can be found in STAR Methods. Schematic diagrams of heat transfer from the heat source of quartz glass surface both in air and in rapeseed oil are shown in Figures 6A and 6B. The temperature probe is positioned as [15μm,0,0] at a specific coordinate within the experimental setup in Figure 1B. Time t dependence of the probe temperature T of quartz glass both in air and in rapeseed oil is shown in Figures 6C and S4. When the laser starts etching in the air, the probe temperature reaches 5,813.2K. As the laser scans along an annular path, the probe temperature drops to 2,128.5K with a decrease of 63.38% at t=0.96ms. When the laser starts etching in the rapeseed oil, the probe temperature reaches 4,773.5K, lower than that in air. Then the probe temperature drops to 2,011.1K with a decrease of 57.87% at t=0.93ms. It takes approximately 5ms to scan a whole circular ring by laser. The temperature is about 4,800∼6,000K near the etching area while the temperature is about 1,000K in the etched area because of temperature dissipation. Simulation results indicate that rapeseed oil contributes to the dissipation of the laser energy, which helps reduce the temperature of the quartz glass surface and decrease the probability of micro-cracks, shown in Figure S4. As the number of scanning times increases, these effects facilitate greater amounts of laser intensity reaching the quartz, as observed in Figures 3C, 3F, 4C, and 4F. During the etching process, the laser focuses on the surface of the quartz glass, causing the etched area of the quartz glass to reach a molten state. As the number of laser scans increases, energy is continuously provided. If the heat from the quartz glass surface is not dissipated in time, it accumulates and is added to the next scan, resulting in a thermal accumulation effect. Because the thermal conductivity of air is relatively low, the temperature on the sample surface is difficult to spread, leading to a thermal accumulation effect and an increased likelihood of micro-cracks. In contrast, rapeseed oil has a higher thermal conductivity and can absorb a significant amount of heat through evaporation, quickly dissipating the heat from the quartz glass surface. Therefore, compared to air, rapeseed oil can effectively reduce the temperature of the upper surface of the quartz glass during the etching intervals, decrease the temperature gradient, and make the temperature field more uniform. This reduces the residual heat and stress, allowing more laser energy to interact with the quartz glass. This suggests that the utilization of rapeseed oil in laser etching not only mitigates the thermal stress on the quartz glass but also minimizes the formation of micro-cracks, which are common in air etching due to rapid temperature fluctuations.

## Discussion

This paper systematically investigates the effects of etching quartz glass microgrooves in air versus rapeseed oil. Preliminary results indicate that rapeseed oil mitigates bubble-related issues and facilitates debris removal through micro-jets. With the increasing scanning times N, the depth of quartz glass microgroove etched in rapeseed oil is deeper than that in the air. In addition, under the conditions of $E_{p}=7.1\;\mu J$, v=50mm/s,N=20, the aspect ratio of the microgroove etched in 58μm rapeseed oil is 1.69, which is greater than that of the microgroove etched in air. According to the ionization model with the Drude equation, we found that the laser intensity to reach the damage threshold of quartz in rapeseed oil is lower than that in air. Based on Fourier heat conduction theory, rapeseed oil can effectively reduce the temperature gradient of the quartz glass. These conclusions will be important for modern industrial manufacturing in etching transparent and hard material, which offers wide prospects for high-precision manufactured optical sensors and photonic devices.

### Limitations of the study

We recognize a limitation in our methodology regarding the precision of oil film thickness control. Our setup might not accurately manage liquid layer uniformity or thickness. Precise control over this parameter is pivotal for a detailed analysis of how oil film thickness influences etching outcomes. We are aware of the necessity for a structured liquid selection process. Upcoming research will delve into the performance of various liquids, questioning if higher viscosity is advantageous or if an alternative liquid with superior etching characteristics at varying viscosities exists. A thorough evaluation could reveal superior etching mediums, bolstering the validity of our study.

## Resource availability

### Lead contact

Further information and requests for resources and reagents should be directed to and will be fulfilled by the lead contact, Jing Liu (jingliu@scuec.edu.cn).

### Materials availability

This study did not generate new unique reagents.

### Data and code availability

• All data reported in this paper will be shared by the lead contact upon request.
• This paper does not report original code.
• Any additional information required to reanalyze the data reported in this paper is available from the lead contact upon request.

## Acknowledgments

This research was supported by the 10.13039/501100001809National Natural Science Foundation of China (no. 11804399) and the Fund for Academic Innovation Teams of South-Central Minzu University (grant number: XTZ24002).

## Author contributions

J.L.: conceptualization, formal analysis, resources, supervision, writing – original draft, and project administration. Y.F.: data curation, formal analysis, resources, and writing – original draft. M. Zheng: conceptualization and writing – original draft. S.C.: validation and data curation. P.W.: validation and data curation. M. Zhao: writing – original draft. Z.H.: conceptualization, methodology, formal analysis, investigation, and writing – original draft. M.L.: conceptualization, methodology, formal analysis, investigation, and writing – original draft.

## Declaration of interests

The authors declare no competing interests.

## STAR★Methods

### Key resources table

**Table:** 

REAGENT or RESOURCE	SOURCE	IDENTIFIER
**Chemicals, peptides, and recombinant proteins**		

Quartz glass	Fujian Ultra Photonics Co., Ltd.	https://www.u-photonics.com/
Rapeseed oil	Shandong Luhua Group Co., Ltd.	https://www.luhua.cn/

**Software**		

Origin 2024	Originlab	https:/www.originlab.com
MATLAB R2022b	MathWorks	https://www.mathworks.com/
ANSYS 2022R1	ANSYS	https://www.ansys.com/

### Method details

#### Experimental equipment and sample

Figure S1 (supplemental information) shows the experimental setup schematic for laser ecthing on the quartz glass surface. The experiments were performed on the laser processing system scanning galvanometer with the femtosecond laser (Newport Corporation SPIRIT 16-HE-SHG, wavelength 1040nm, pulse duration 388fs, pulse repetition rate 100kHz). The focal length of field lens is 100mm. The focusing spot diameter is about 14μm. The experimental sample is a square piece of JGS1 quartz glass. The sample size is $20\times 20\times 1\;{\text{mm}}^{3}$. Its melting point is 2000K and its refractive index is 1.45. Without any prior treatment, 0.02ml of rapeseed oil was directly applied to the surface of the JGS1 quartz glass using a needle. The morphology and depth inspection equipment is a VK-X250 focused microscope manufactured by KEYENCE, Japan.

#### Heat source models

The Gaussian laser beam as a heat source diffuses into the interior of the quartz glass and the surrounding environment (rapeseed oil or air). The moving path of the heat source in the simulation is consistent with the experiment, shown in Figure 1B. In the etching process, the initial position of the micro-groove is $\left(x_{0},y_{0},z_{0}\right)=\left(0,0,0\right)$. The heat source position (x,y,z) can be defined as $x=D\;\cos\left(\omega t\right)/2+x_{0}$, $y=D\;\sin\left(\omega t\right)/2+x_{0}$, $z=z_{0}$. Here D=30μm. And ω=2πvt, v is the scanning speed, and t is the scanning time. A Gaussian internal heat source $Q=Ce^{-\frac{\left[{\left(x-x_{0}\right)}^{2}+{\left(y-y_{0}\right)}^{2}+{\left(z-z_{0}\right)}^{2}\right]}{d^{2}/4}}$. And $C=\frac{\left(I_{0}-I\right)\cdot t\cdot f}{\pi \left({\left(D+d\right)}^{2}-{\left(D-d\right)}^{2}\right)/4}$ is the power density of the heat source, where the repetition frequency f=100KHz, d=14μm. Based on the Lambert-Beer’s law, the exit light intensity is $I=I_{0}\cdot e^{-\alpha L_{quartz}}$, where $I_{0}$ is the incident light intensity, and $\alpha =0.01\;{\text{cm}}^{-1}$ is the absorption coefficient of quartz glass.

According to Fourier heat conduction theory, the transient heat conduction differential equation can be written by $\kappa \left(\frac{\partial^{2}T}{\partial x^{2}}+\frac{\partial^{2}T}{\partial y^{2}}+\frac{\partial^{2}T}{\partial z^{2}}\right)+Q=c\rho \frac{\partial T}{\partial t}$, where ρ is the density of the material, c is the specific heat capacity, κ is the thermal conductivity, T is the spatial distribution function of quartz glass temperature field, and Q is the heat source. The physical parameters are shown in Table S2 (supplemental information). It is worth noting that the specific heat capacity of rapeseed oil varies with temperature change, which can be expressed as $c_{oil}=2.2379+0.00723T-1.2619\times 10^{-5}T^{2}$.

#### Initial condition and boundary condition

The Gaussian laser heat source is loaded on the upper surface of the quartz glass in the form of heat flux density. Therefore, the convective heat transfer between the rapeseed oil and the quartz glass can be expressed as $\kappa \partial T/\partial n-Q+\sigma C_{s}\left(T^{4}-T_{0}^{4}\right)=0$ .where ∂T/∂n is the temperature gradient along n, $\sigma =5.67\times 10^{-8}\;W/\left(m^{2}\cdot k^{4}\right)$ is Stefan-Boltzmann constant, $C_{s}=0.836\;W/\left(m^{2}\cdot k\right)$ is the thermal radiation coefficient, and the initial temperature $T_{0}=295\;K$. The mesh size around the simulation path is controlled by encryption processing, and the minimum size of the grid is set to 2μm. The power density of the Gaussian heat source is $1592\;W/{\text{mm}}^{2}$ and the moving speed is 20mm/s.

### Quantification and statistical analysis

The Origin2024 was used for statistical analysis. The evolution of the electron density was calculated using MATLAB R2022b. The thermal source model calculation of the quartz glass temperature was performed using ANSYS 2022R1.
