# Supplementary material for: Ultra-thin rapeseed oil-assisted femtosecond laser etching on quartz glass microgrooves
Source: iScience. 2025 Feb 1;28(2):111921. doi: 10.1016/j.isci.2025.111921 (PMC11869957; doi:10.1016/j.isci.2025.111921)
Supplement: Document S1. Figures S1–S4 and Tables S1 and S2 [file mmc1.pdf]

iScience, Volume *28*

Supplemental information

Ultra-thin rapeseed oil-assisted femtosecond laser etching on quartz glass microgrooves

Jing Liu, Yimin Feng, Mian Zheng, Shangkai Chen, Pengran Wang, Mengdan Zhao, Zhaoxian Huang, and Ming Li

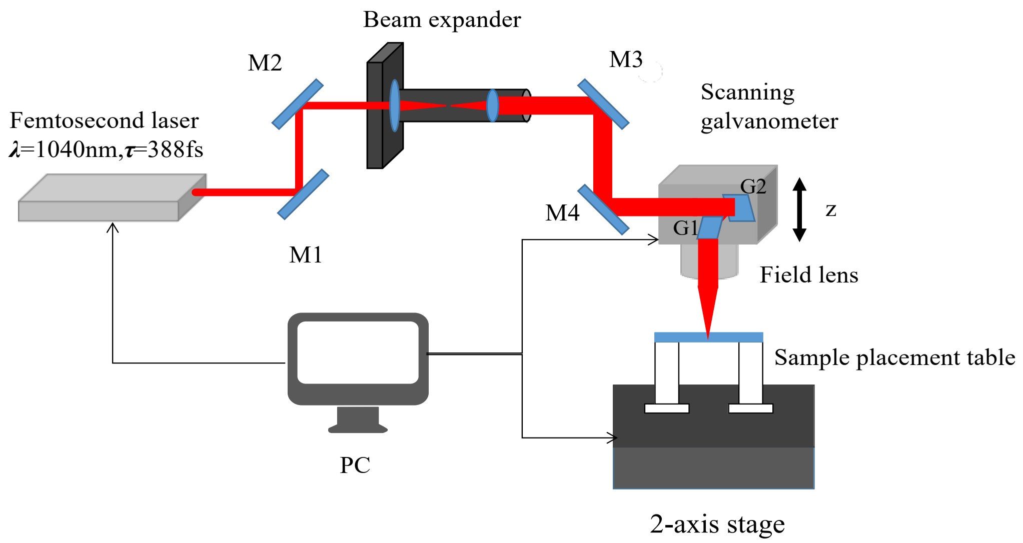

Figure S1. Experimental setup schematic for laser ecthing on the quartz glass surface.

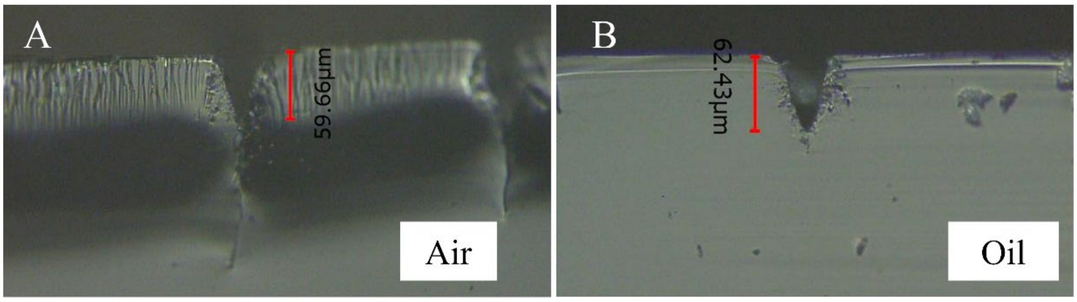

Figure S2. When $E_p = 7.1\ \mu J,\ v = 50\ mm/s,\ N = 5$, the cross-sectional morphology of quartz glass microgrooves in air and rapeseed oil.

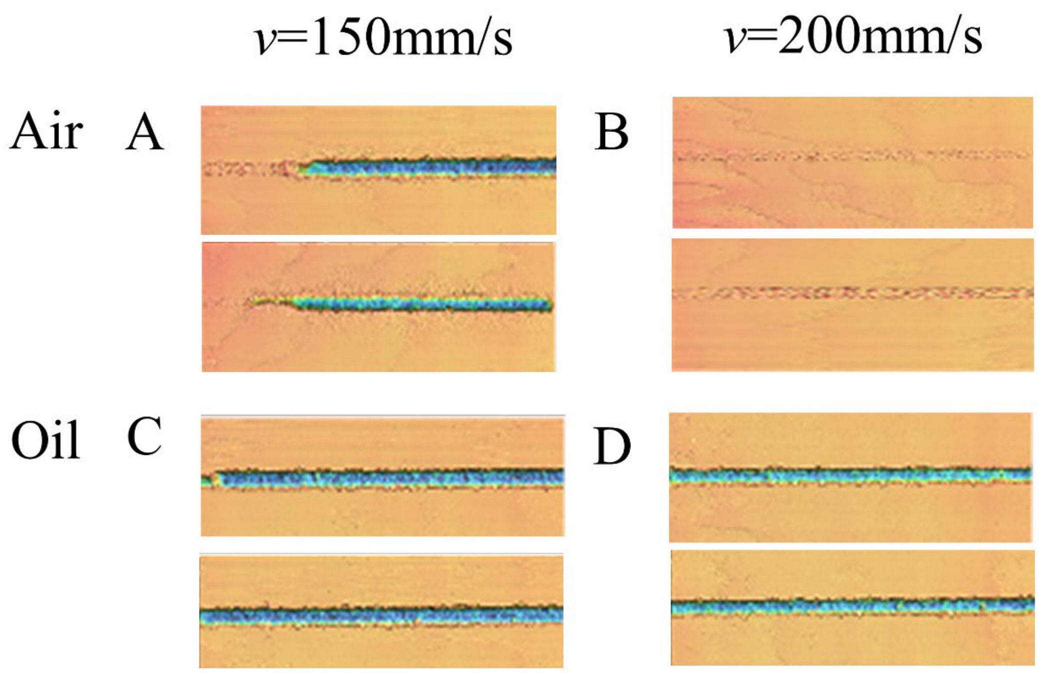

Figure S3. When $E_p = 7.1\ \mu\text{J}$, $N = 1$, the surface morphology of quartz glass microgrooves in air and in rapeseed oil. In air, (A) $v = 150\ \text{mm/s}$ (B) $v = 200\ \text{mm/s}$. In rapeseed oil, (C) $v = 150\ \text{mm/s}$ (D) $v = 200\ \text{mm/s}$.

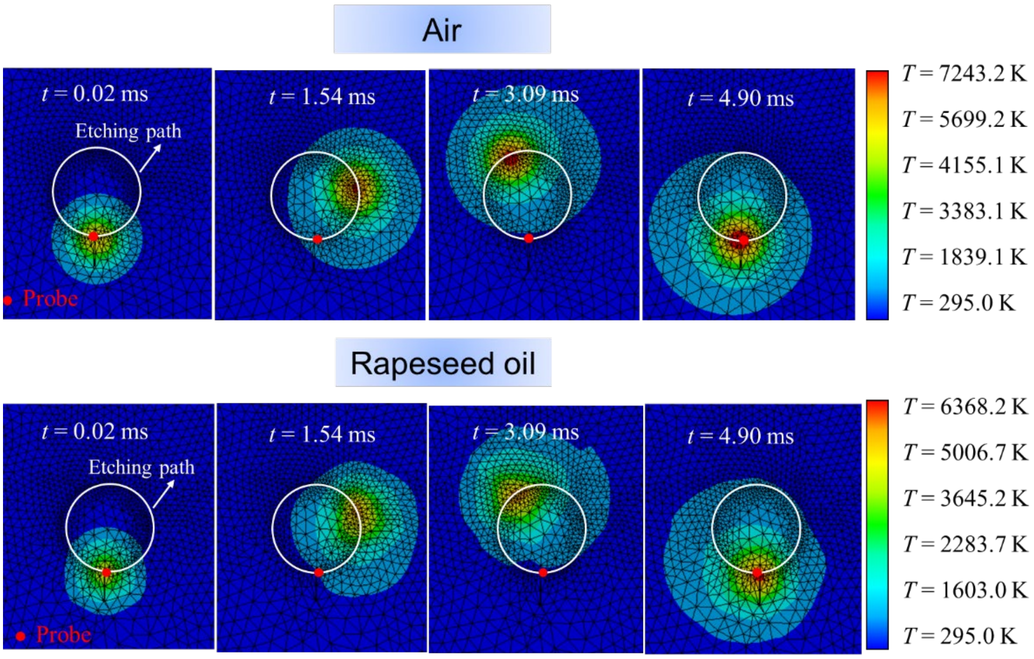

Figure S4. The temperature distribution of quartz glass both in air and in rapeseed oil at t = 0.02 ms, 1.54 ms, 3.09 ms and 4.90 ms respectively. The temperature probe is located at the position $[15\ \mu\text{m},\ 0,\ 0]$.

Table S1 Main parameters used in numerical analysis.

Description	Unit	Value
SiO_2 bandgap Δ_q	eV	8.9
Rapeseed oil bandgap Δ_o	eV	3.5
Electron mass m_e	kg	9.11×10^{-31}
Effective electron mass of SiO_2 m_e^*	kg	0.3 m_e
Hole mass of SiO_2 m_h	kg	0.58 m_e
Electron charge e	C	1.60×10^{-19}
Vacuum dielectric permittivity ε_0	F/m	8.85×10^{-12}
Plank constant h	eVs	6.63×10^{-34}
Speed of light c	m/s	3×10^{8}
Electron saturation velocity v_s	cm/s	2×10^{7}
Collision scattering field E_I	MV/cm	30
Photons scattering field E_P	MV/cm	3.2
Thermal scattering field E_{KT}	MV/cm	0.01
Free electron relaxation time τ_e	fs	1
Laser wavelength λ	nm	1040
Laser pulse duration t_p	fs	388
The radius of the Laser beam r_0	μm	~7

Table S2. Physical parameters in femtosecond laser etching of quartz glass.

Parameters	Unit	Value
Incident light power I_0	w	0.71
Thermal conductivity of rapeseed oil κ_{oil}	w/(m·K)	0.2
Thermal conductivity of quartz glass κ_{quartz}	w/(m·K)	1.34
Thermal conductivity of air κ_{air}	w/(m·K)	0.02
Heat capacity of rapeseed oil c_{oil}	J/(kg·K)	2597
Heat capacity of quartz c_{quartz}	J/(kg·K)	892
Heat capacity of air c_{air}	J/(kg·K)	1005
Absorption coefficient of quartz glass α	cm^{-1}	0.01
Quartz glass thickness L_{quartz}	μm	10^3
Oil thickness L_{oil}	μm	58
Density of rapeseed oil ρ_{oil}	g/cm^3	0.91
Density of quartz glass ρ_{quartz}	g/cm^3	2.2
Density of air ρ_{air}	g/cm^3	1.29×10^{-3}
Room temperature T_0	K	295
Boiling temperature of rapeseed oil T_{oil}	K	610
